# Cryptococcal Meningitis Screening and Community-based Early Adherence Support in People With Advanced Human Immunodeficiency Virus Infection Starting Antiretroviral Therapy in Tanzania and Zambia: A Cost-effectiveness Analysis

**DOI:** 10.1093/cid/ciz453

**Published:** 2019-05-31

**Authors:** Godfather Dickson Kimaro, Lorna Guinness, Tinevimbo Shiri, Sokoine Kivuyo, Duncan Chanda, Christian Bottomley, Tao Chen, Amos Kahwa, Neil Hawkins, Peter Mwaba, Sayoki Godfrey Mfinanga, Thomas S Harrison, Shabbar Jaffar, Louis W Niessen

**Affiliations:** 1 Muhimbili Medical Research Centre, National Institute of Medical Research, Dar es Salaam, United Republic of Tanzania; 2 Department of Infectious Disease Epidemiology, London School of Hygiene and Tropical Medicine, United Kingdom; 3 Department of Global Health and Development, London School of Hygiene and Tropical Medicine, United Kingdom; 4 Department of International Public Health, Liverpool School of Tropical Medicine, United Kingdom; 5 University Teaching Hospital, Lusaka Apex Medical University, Zambia; 6 Department of Internal Medicine and Directorate of Research and Postgraduate Studies, Lusaka Apex Medical University, Zambia; 7 Institute for Infection and Immunity, Centre for Global Health, St George’s University of London, United Kingdom; 8 Department of International Health, Johns Hopkins Bloomberg School of Public Health, Baltimore, Maryland

**Keywords:** cost-effectiveness, HIV late-stage disease, cryptococcal meningitis, adherence support, Africa

## Abstract

**Background:**

A randomized trial demonstrated that among people living with late-stage human immunodeficiency virus (HIV) infection initiating antiretroviral therapy, screening serum for cryptococcal antigen (CrAg) combined with adherence support reduced all-cause mortality by 28%, compared with standard clinic-based care. Here, we present the cost-effectiveness.

**Methods:**

HIV-infected adults with CD4 count <200 cells/μL were randomized to either CrAg screening plus 4 weekly home visits to provide adherence support or to standard clinic-based care in Dar es Salaam and Lusaka. The primary economic outcome was health service care cost per life-year saved as the incremental cost-effectiveness ratio (ICER), based on 2017 US dollars. We used nonparametric bootstrapping to assess uncertainties and univariate deterministic sensitivity analysis to examine the impact of individual parameters on the ICER.

**Results:**

Among the intervention and standard arms, 1001 and 998 participants, respectively, were enrolled. The annual mean cost per participant in the intervention arm was US$339 (95% confidence interval [CI], $331–$347), resulting in an incremental cost of the intervention of US$77 (95% CI, $66–$88). The incremental cost was similar when analysis was restricted to persons with CD4 count <100 cells/μL. The ICER for the intervention vs standard care, per life-year saved, was US$70 (95% CI, $43–$211) for all participants with CD4 count up to 200 cells/μL and US$91 (95% CI, $49–$443) among those with CD4 counts <100 cells /μL. Cost-effectveness was most sensitive to mortality estimates.

**Conclusions:**

Screening for cryptococcal antigen combined with a short period of adherence support, is cost-effective in resource-limited settings.

## Teaser

In a large phase 3 randomized controlled trial among persons presenting with late-stage HIV infection, we showed that cryptococcal meningitis screening and community-based early adherence support was a highly cost-effective strategy in reducing all-cause mortality.

Antiretroviral therapy (ART) is available widely in Africa, but human immunodeficiency virus (HIV)–associated mortality remains high, particularly during the first few months that patients come into care [1–4]. Most of the early mortality is attributed to tuberculosis and cryptococcal meningitis [5–8].

We recently conducted a large randomized controlled trial among HIV-infected patients starting ART with CD4 count <200 cells/μL in Dar es Salaam, Tanzania and in Lusaka, Zambia, known as the Reduction of Early Mortality Among HIV-infected Subjects Starting Antiretroviral Therapy (REMSTART) trial [9]. The intervention comprised (1) screening for cryptococcal infection using serum cryptococcal antigen (CrAg) combined with preemptive fluconazole therapy for patients testing CrAg positive and (2) weekly home visits for the first 4 weeks for patients on ART by trained lay workers to provide adherence support. This package was compared with standard care. Participants in both arms were screened for tuberculosis with sputum Xpert assay irrespective of symptoms and all were initiated rapidly on ART within a median of 14 days (interquatile range, 9–20 days) following presentation. At 12 months of follow-up, all-cause mortality was 28% (95% confidence interval [CI], 11%–43%) lower in the intervention arm compared to that in the standard care arm.

Findings from the trial informed the World Health Organization (WHO) guidelines on the management of late-stage HIV infection in Africa [9]. However, coverage of CrAg screening remains low [10] despite the WHO recommendation and low cost of a CrAg test, and very few settings offer repeated initial adherence support to patients with advance disease starting ART. To inform policy and practice considerations, we conducted a cost-effectiveness analysis of CrAg screening and early adherence support compared with standard care using data from the REMSTART trial.

## METHODS

### Study Design and Participants

The REMSTART trial results have been published [9, 11]. In brief, the participants were HIV-infected adults presenting for care at routine health services between February 2012 and September 2013. A total of 1001 patients were randomized to the intervention arm and 998 to standard care.

In the intervention arm, the participants were screened for serum CrAg using lateral flow tests (IMMY, Norman, Oklahoma) and promptly started on fluconazole (800 mg per day for 2 weeks followed by 400 mg per day for 8 weeks) if they were CrAg positive. In addition, they received up to 4 weekly home visits by trained lay workers during the first 4 weeks of starting ART. The primary endpoint was all-cause mortality at 12 months of follow-up.

The economic evaluation was nested within the trial and done from a healthcare provider’s perspective [11, 12]. The resource use and related costs were collected from all participants. Unit costs were obtained from Dar es Salaam [10]. We assessed the incremental cost-effectiveness ratio (ICER) of the intervention vs standard treatment for the duration of the follow-up period. It was assumed that the illness pathways of the control and treatment group would be equivalent thereafter and that the incremental impact of the intervention on morbidity would thus be negligible.

### Measurement of Costs and Outcomes

Health cost data were collected in 2012 and capital costs were discounted using a discount rate of 7.8% equal to the 2-year Tanzanian government bond at the time of calculation, June 2014 [13]. All costs were converted to 2017 US dollars using a gross domestic product deflator and an exchange rate [14].

Life-years gained was used as the primary outcome measure. As the morbidity effects of the intervention relative to the mortality effect are likely to be minimal in this context, these can be used to approximate Disability Adjusted Life Years saved. To estimate life-years gained, we computed the difference in the annual probability of death between the intervention and control arms (rather than the incidence rates of death per person-years of observation used in the main paper [9]) and multiplied this by the related life expectancy. We assumed that average life expectancy was 24.0 years (95% CI, 22.2–25.8 years) for all patients with CD4 count up to 200 cells/μL 18.7 years (95% CI, 17.2–20.3 years) for patients with CD4 cell count <100 cells/μL based on data for similar patients on ART [15].

### Statistical Analyses

Differences between the intervention and standard care in costs and mortality were estimated using seemingly unrelated regression equations to account for the correlation between costs and mortality [16]. The models included baseline body weight, baseline CD4 cell count, and baseline hemoglobin levels, which were shown to predict mortality at 12 months. ICERs were estimated by dividing mean incremental costs by the mean number of life-years saved, and cost-effectiveness planes and acceptability curves were plotted. Confidence intervals were obtained using nonparametric bootstrapping.

Univariate deterministic sensitivity analyses were done to assess the impact of parameter uncertainty on the ICER and were presented as a standard tornado sensitivity graph. The parameter ranges used for sensitivity analysis were based on 95% CIs calculated from the REMSTART data and the observed life expectancy CIs [15]. We conducted a secondary analysis, restricting to participants with CD4 count level <100 cells/μL, as CrAg screening is recommended for this HIV-infected population [17].

### Ethics Statement

The study was approved by the ethics committee of the London School of Hygiene and Tropical Medicine, the Ethics and Research Science Committee in Zambia, and the National Health Research Ethics Subcommittee in Tanzania.

## RESULTS

Table 1 presents the unit costs for the different components of ART services and the quantity of resources utilized by participants. The mean cost of a home visit was almost 3 times higher than the cost of the participant visiting the health facility. The unit price for the home visit comprised monthly salary for the lay worker, training, communication, and transport allowance. CrAg testing was about a quarter of the cost of either a CD4 cell count or Xpert test.

**Table 1.** Unit Costs (in 2017 US Dollars) and Quantity of Resources Utilized per Study Participant, by Arm, Over 12 Months^a^

Cost Component	Unit Price, US$	Unit of Measurement	Intervention (n = 1001), Mean (SD)	Standard Care (n = 998), Mean (SD)
Outpatient visits				
Initial visit	8.93	Visit	1.05 (0.36)	1.04 (0.37)
ART eligibility assessment visit	7.99	Visit	1.16 (0.44)	1.10 (0.33)
Biannual clinic review visit	8.92	Visit	0.81 (0.77)	0.79 (0.81)
Routine follow-up visit	7.62	Visit	3.89 (2.77)	3.56 (2.81)
Home visit^b^	19.51	Visit	3.06 (1.43)	0.01 (0.18)
Laboratory				
CD4 count test	20.86	Test	1.55 (0.62)	1.51 (0.62)
Liver function (ALT) test	1.16	Test	1.27 (0.59)	1.19 (0.56)
Creatinine test	0.41	Test	0.89 (0.44)	0.87 (0.44)
Hemoglobin test	1.15	Test	1.46 (0.73)	1.35 (0.68)
Syphilis (VDRL) test	1.13	Test	0.03 (0.18)	0.03 (0.17)
Pregnancy test	2.50	Test	0.14 (0.47)	0.13 (0.44)
Xpert test	25.23	Test	0.96 (0.61)	0.82 (0.46)
CrAg test	5.24	Test	0.98 (0.13)	0.01 (0.08)
CSF test	21.55	Test	0.01 (0.09)	0.00 (0.00)
Chest radiograph	2.70	Radiograph	0.05 (0.21)	0.04 (0.19)
Medication				
Days on ART	0.56	Day	260.47 (137.23)	250.27 (140.70)
Days on cotrimoxazole treatment	0.02	Day	263.22 (136.00)	254.82 (139.76)
10-wk fluconazole course^c^	6.35	Course	0.07 (0.37)	0.00 (0.00)
Hospitalization				
Overnight hospital stay	35.00	Day	0.18 (1.17)	0.22 (1.55)

Abbreviations: ALT, alanine aminotransferase; ART, antiretroviral therapy; CrAg, cryptococcal antigen; CSF, cerebrospinal fluid; SD, standard deviation; VDRL, Venereal Disease Research Laboratory.

^a^Unit prices for all tests apart from patients do not include overhead costs.

^b^Home visit costs included monthly salary for the lay worker, communication and transport allowance, and training costs.

^c^800 mg per day for 2 weeks, followed by 400 mg per day for 8 weeks and 200 mg/day thereafter (after 10 weeks).

The total mean costs per patient did not differ by CD4 count category for either arm (Table 2). The intervention resulted in approximately 30% (95% CI, 25%–35%) increase in the cost per patient, and resulted in an ICER of US$70 (95% CI, $43–$211) for participants with CD4 <200 cells/μL.

**Table 2.** Incremental Cost-effectiveness Ratios (Incremental Cost per Life-year Saved) Comparing the Intervention With Standard Care According to Baseline CD4 Cell Count

		Standard Care Arm				Intervention Arm				Incremental Comparison of the Intervention vs Standard Care		
				All-cause Mortality				All-cause Mortality				
CD4 Cell Count	Life Expectancy, y^a^ (95% CI)	No.	Mean Total Cost/Person, US$ (95% CI)	Events (95% CI)	Death Rate (95% CI)	No.	Mean Total Cost/Person, US$ (95% CI)	Events (95% CI)	Death Rate (95% CI)	Incremental Cost/Person^b^, US$ (95% CI)	Incremental Death^b^, % (95% CI)	ICER^c^ (95% CI)
<200 cells/μL	24.0	998	262	180	18.0	1001	339	134	13.4	77	–4.6	70
	(22.2–25.8)		(254–269)	(156–203)	(15.6–20.3)		(331–347)	(113–156)	(11.3–15.6)	(66–88)	(–7.8 to –1.3)	(43–211)
<100 cells/μL	18.7	707	262	144	20.2	724	341	114	15.7	79	–4.5	91
	(17.2–20.3)		(253–271)	(123–165)	(17.4–23.3)		(331–350)	(94–134)	(13.0–18.5)	(65–92)	(–8.6 to –.6)	(49–443)

Abbreviations: CI, confidence interval; ICER, incremental cost-effectiveness ratio.

^a^Weighted life expectancy based on a previous study [15].

^b^Differences were estimated using regression equations adjusting for body weight and hemoglobin levels measured at baseline.

^c^Estimated by dividing incremental cost by the mean number of life-years saved (ie, incremental death multiplied by life expectancy).

The absolute reductions in death rates between the intervention and standard care did not differ significantly by CD4 cell count category (Table 2). The mortality risk ratio of the intervention (134/1001 deaths) vs standard care (180/998 deaths) was 0.74 (95% CI, .60–.91) among all participants with CD4 <200 cells/μL. For participants with a CD4 count <100 cells/μL, this risk ratio was 0.77 (95% CI, .62–.96) (114/724 vs 144/707 deaths).

### Cost-effectiveness: Uncertainty and Sensitivity Analyses

**Figure 1.**
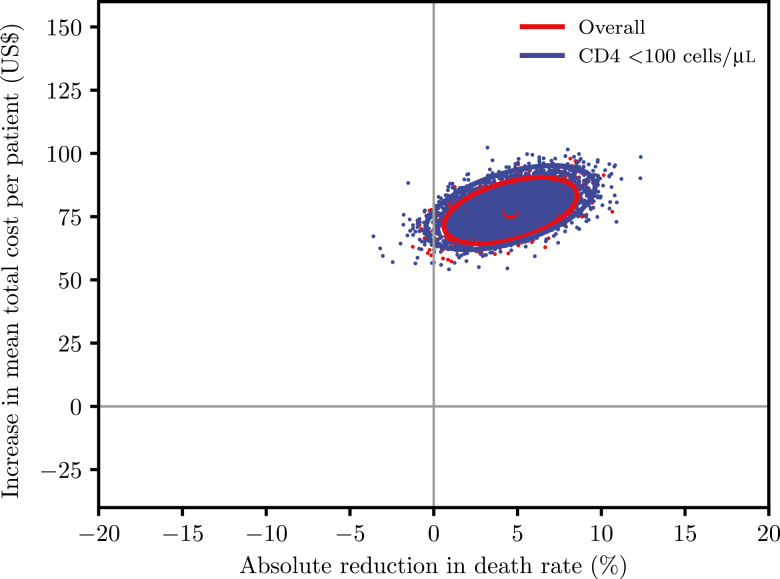
Cost-effectiveness planes after bootstrapping showing uncertainty in the estimated incremental costs and the annual probability of death (%) for persons with CD4 count up to 200 cells/μL (red dots) and among persons with CD4 count < 100 cells/μL (blue dots). Ellipses represent 95% confidence intervals and dots represent estimated incremental costs and death rates.

The intervention was more costly but more effective than the standard care (Figure 1). Average ICERs increased somewhat with decreasing CD4 cell count category (Table 2).

**Figure 2.**
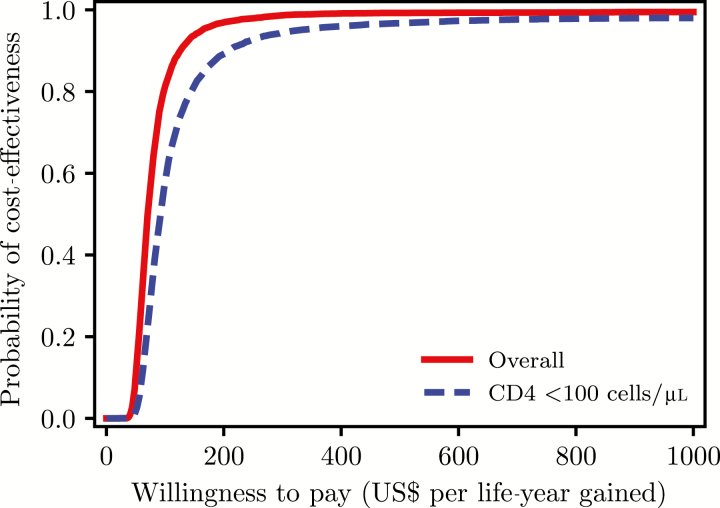
Cost-effectiveness acceptability curves showing the probability of cost-effectiveness at different willingness-to-pay thresholds according to baseline CD4 cell count.

The probability that the intervention is cost-effective when compared with the standard care if the decision makers are willing to pay between US$100 and US$150 for an additional life-year saved varies between 84% and 95% in persons with CD4 count <200 cells/μL and between 62% and 82% in persons with CD4 count <100 cells/μL (Figure 2).

**Figure 3.**
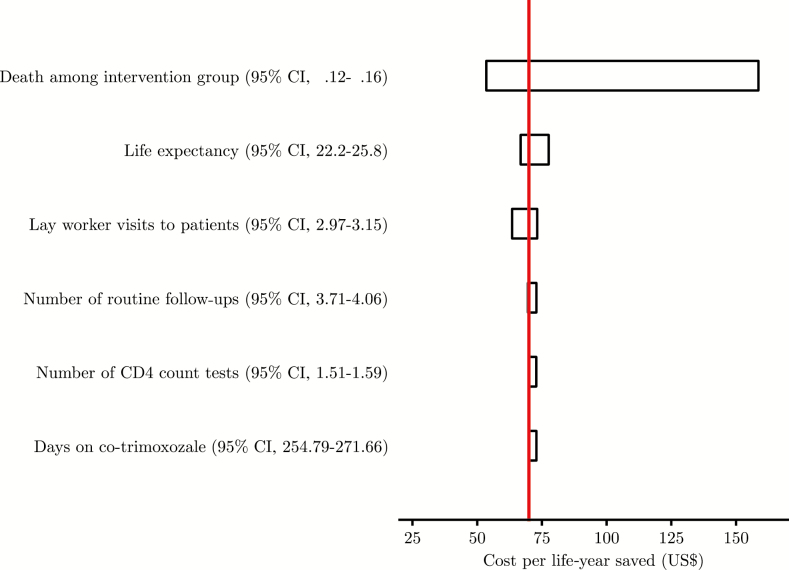
Tornado diagram of change in the base-case incremental cost-effectiveness ratio among persons with CD4 count up to 200 cells/μL produced from a deterministic one-way analysis of 6 input parameters. The ranges used in the sensitivity analysis were based on 95% confidence intervals (CIs) calculated from the Reduction of Early Mortality among HIV-infected Subjects Starting Antiretroviral Therapy (REMSTART) data and previous studies.

In sensitivity analyses, the most influential parameter of the ICER was the mortality rate (Figure 3). If the annual mortality in the intervention arm is as low as 12%, then the ICER is reduced to US$54 per life-year saved.

## DISCUSSION

Our analysis demonstrates that screening of patients presenting with advanced HIV disease for cryptococcal meningitis combined with a short intensive period of adherence support is likely to be cost-effective for most settings in Africa [18–20]. The average cost per patient of the intervention compares favorably with those of other interventions in Africa such as providing outpatient HIV counseling, testing, and ART treatment in Nigeria [21], and other ART services in Zambia, Kenya, Burkina Faso, and South Africa [22–27]. A strength of the trial is that it was done in close to normal health service conditions with, for example, government-employed healthcare workers managing the patients using national guidelines.

The home-based adherence support component involved the use of trained lay workers visiting the homes of patients by public transport. The cost of home visits—up to about $80 for most participants—was the major driver of total costs. Trial participants were scattered and lay workers traveled between patient houses. In a small subsample, we estimated that the lay workers took about 50–60 minutes to travel from one patient’s house to the next, and that this was about twice the amount of time that they actually spent at a patient’s home. Furthermore, on some days, particularly near the start of the trial, some lay workers made only 1 or 2 home visits a day because we had not recruited sufficient numbers of patients. In real life, if this approach was rolled out universally, many patients would be on therapy and the increased patient density would reduce travel distances. Health services would also identify long-term, lower-cost ways of traveling between houses, such as field workers traveling on bicycles or motorbikes, which were not practical during the normal course of trial. The costs could be reduced further by use of alternative methods of providing support, such as through mobile phones. If the cost per home visit decreased from $19.51 to $5, this will result in an average incremental cost of $33 for the intervention, subsequently reducing the average ICER to $30 per life-year saved, using a conservative life expectancy of 24 years. If we assume a life expectancy of 35 years for participants on ART, the ICER becomes $20 per life-year saved.

In the CrAg screening component, we have overestimated the cost of CrAg testing. The cost of a single test was $5 and a single test was done to identify a single CrAg-positive person. The CrAg test is simple and its cost will fall with expanded use. Also, the prevalence of CrAg positivity was lower in our study than the average reported in Africa [17, 28].

Finally, we calculated cost-effectiveness based on a healthcare perspective and did not assess societal benefits, for example, the benefits of increased productivity arising from improved health status. We also used a conservative estimate of life expectancy, calculated taking CD4 cell count into account. Had we used WHO estimates of life expectancy for Tanzania [29], the ICER would have been even lower.

Thus, all in all, the intervention tested will be highly cost-effective, with a likely ICER of somewhere between $10 and $20 per life-year saved.

With complex interventions, as that employed here, it is impossible to disentangle the effects of the CrAg individual component from the enhanced adherence component, but it is very clear that, as combined, this is an important new intervention for African HIV services to consider for persons with advanced HIV disease. The effects of CrAg screening beyond CD4 count of ≥100 cells/μL are unknown as there are no other data, besides ours, that have tested for CrAg at higher than this CD4 count. Our study found fewer CrAg-positive cases in the group that had CD4 counts between 100 and 200 cells/μL, and as expected, the death rate in this group was lower than in those with lower CD4 counts. This lowers the ICER. In our study, the combined intervention was as cost-effective at CD4 count <200 cells/μL as at CD4 count <100 cells/μL, presumably because survival is better achieved when we initiate interventions at an earlier disease stage. More data are needed of CrAg prevalence among CD4 counts >100 cells/μL.

Because of the limited evidence around cost-effectiveness, coverage of CrAg screening combined with preemptive fluconazole has remained low [10], despite being recommended by WHO [9]. A study from South Africa had suggested that such an intervention is cost-effective [30], but the findings from a middle-income setting are difficult to generalize to low-resource settings. In Uganda, a similar cost-effectiveness study led to the same conclusions [31]. Both studies had modeled cost-effectiveness from the healthcare perspective, and have not been enough to facilitate a higher scale-up of CrAg screening. Our study estimated cost-effectiveness using empirical data from a large cohort in 2 countries. We calculated costs from the healthcare perspective, and the findings demonstrate clearly that this intervention is highly cost-effective even in low-resource settings.

For patients who are CrAg positive, mortality is 2–3 times higher even with prompt preemptive treatment with fluconazole and combined with enhanced adherence support than that in CrAg negatives [9]. A more potent regimen is needed. A recent large trial demonstrated that flucytosine, when added to fluconazole, is a highly effective oral combination for the management of patients with confirmed cryptococcal meningitis and, even at current prices, is highly cost-effective compared with fluconazole monotherapy, with an ICER of US$65 per life-year saved [32]. This oral combination needs urgent evaluation of cost-effectiveness in CrAg-positive persons. Findings from Uganda [33] and South Africa [34] suggest that in asymptomatic persons, the CrAg titer predicts mortality, suggesting that the oral combination might only be needed for this group.

The number of persons presenting with advanced disease is likely to remain high for the foreseeable future, as alongside the large number of people who still delay accessing ART services, there are many dropping out of care or failing on ART. With the findings of this analysis and the recent trial of the management of cryptococcal meningitis [5], we can now begin to reduce this mortality in cost-effective and sustainable ways.

## Notes

***Author contributions.*** S. J., S. G. M., and T. S. H. designed the original trial and S. G. M. led the coordination of the trial with support from S. K., D. C., and P. M.; C. B. did the original trial efficacy analysis, which was also used in the present manuscript. G. D. K. and L. G. designed the health economics study nested within the trial; G. D. K., with support from S. K. and D. C., coordinated the health economics data collection. G. D. K. wrote the first draft of the manuscript and played the major role in writing thereafter with regular support from L. W. N., L. G., and S. J.; T. S., S. K., C. B., A. K., N. H., S. G. M., and T. S. H. commented on several drafts. G. D. K. led the initial analysis of costs and cost-effectiveness and oversaw and contributed to the final cost-effectiveness analysis, which was led by T. S. and T. C. The analysis was supervised initially by L. G. and then by L. W. N. All authors contributed to the near final drafts of the manuscript and read and approved the final draft. The corresponding author had full access to all the data in the study and had the final responsibility for the decision to submit for publication.

***Acknowledgments.*** The authors thank the patients and the many healthcare providers who participated and made the study possible, and the public health services in Dar es Salaam and Lusaka for their guidance and support.

***Disclaimer.*** The funders of the study had no role in study design, data collection, data analysis, data interpretation, or writing of the report.

***Financial support.*** This project is part of the European and Developing Countries Clinical Trials Partnership 2 (EDCTP2) program supported by the European Union (grant number IP.2009.33011.003 -REMSTART).

***Potential conflicts of interest.*** The authors: No reported conflicts of interest. All authors have submitted the ICMJE Form for Disclosure of Potential Conflicts of Interest. Conflicts that the editors consider relevant to the content of the manuscript have been disclosed.

## References

[1] Amuron B, Namara G, Birungi J, et al. Mortality and loss-to-follow-up during the pre-treatment period in an antiretroviral therapy programme under normal health service conditions in Uganda. BMC Public Health 2009; 9:290. 19671185 10.1186/1471-2458-9-290 PMC2734853

[2] Kranzer K, Govindasamy D, Ford N, Johnston V, Lawn SD Quantifying and addressing losses along the continuum of care for people living with HIV infection in sub-Saharan Africa: a systematic review. J Int AIDS Soc 2012; 15:17383. 23199799 10.7448/IAS.15.2.17383 PMC3503237

[3] Lawn SD, Harries AD, Anglaret X, Myer L, Wood R Early mortality among adults accessing antiretroviral treatment programmes in sub-Saharan Africa. AIDS 2008; 22:1897–908. 18784453 10.1097/QAD.0b013e32830007cd PMC3816249

[4] Rosen S, Fox MP Retention in HIV care between testing and treatment in sub-Saharan Africa: a systematic review. PLoS Med 2011; 8:e1001056. 21811403 10.1371/journal.pmed.1001056 PMC3139665

[5] Molloy SF, Kanyama C, Heyderman RS, et al; ACTA Trial Study Team Antifungal combinations for treatment of cryptococcal meningitis in Africa. N Engl J Med 2018; 378:1004–17. 29539274 10.1056/NEJMoa1710922

[6] Jarvis JN, Meintjes G, Williams A, Brown Y, Crede T, Harrison TS Adult meningitis in a setting of high HIV and TB prevalence: findings from 4961 suspected cases. BMC Infect Dis 2010; 10:67. 20230635 10.1186/1471-2334-10-67 PMC3161361

[7] Rajasingham R, Smith RM, Park BJ, et al. Global burden of disease of HIV-associated cryptococcal meningitis: an updated analysis. Lancet Infect Dis 2017; 17:873–81. 28483415 10.1016/S1473-3099(17)30243-8 PMC5818156

[8] Durski KN, Kuntz KM, Yasukawa K, Virnig BA, Meya DB, Boulware DR Cost-effective diagnostic checklists for meningitis in resource-limited settings. J Acquir Immune Defic Syndr 2013; 63:e101–8. 23466647 10.1097/QAI.0b013e31828e1e56 PMC3683123

[9] Mfinanga S, Chanda D, Kivuyo SL, et al; REMSTART Trial Team Cryptococcal meningitis screening and community-based early adherence support in people with advanced HIV infection starting antiretroviral therapy in Tanzania and Zambia: an open-label, randomised controlled trial. Lancet 2015; 385:2173–82. 25765698 10.1016/S0140-6736(15)60164-7

[10] Pac L, Horwitz MM, Namutebi AM, et al. Implementation and operational research: integrated pre-antiretroviral therapy screening and treatment for tuberculosis and cryptococcal antigenemia. J Acquir Immune Defic Syndr 2015; 68:e69–76. 25761234 10.1097/QAI.0000000000000527 PMC4357272

[11] Kimaro GD, Mfinanga S, Simms V, et al; REMSTART Trial Team The costs of providing antiretroviral therapy services to HIV-infected individuals presenting with advanced HIV disease at public health centres in Dar es Salaam, Tanzania: findings from a randomised trial evaluating different health care strategies. PLoS One 2017; 12:e0171917. 28234969 10.1371/journal.pone.0171917 PMC5325220

[12] Frick KD Microcosting quantity data collection methods. Med Care 2009; 47:S76–81. 19536026 10.1097/MLR.0b013e31819bc064 PMC2714580

[13] Bank of Tanzania. The Treasury Bond. Dar es Salaam: United Republic of Tanzania, 2012 Available at: http://www.bot-tz.org/FinancialMarkets/TBonds/TBondCallforTender/2012-JUNE-13-TBOND.pdf. Accessed 14 November 2018.

[14] World Bank. GDP deflator. Available at: http://data.worldbank.org/indicator/NY.GDP.DEFL.KD.ZG. Accessed 14 November 2018.

[15] Mills EJ, Bakanda C, Birungi J, et al. Life expectancy of persons receiving combination antiretroviral therapy in low-income countries: a cohort analysis from Uganda. Ann Intern Med 2011; 155:209–16. 21768555 10.7326/0003-4819-155-4-201108160-00358

[16] Zellner A An efficient method of estimating seemingly unrelated regression equations and tests for aggregation bias. J American Stat Assoc 1962; 57:348–68.

[17] Ford N, Shubber Z, Jarvis JN, et al. CD4 cell count threshold for cryptococcal antigen screening of HIV-infected individuals: a systematic review and meta-analysis. Clin Infect Dis 2018; 66:152–9. 10.1093/cid/cix1143 PMC5850628 29514236

[18] Horton S Cost-effectiveness analysis in disease control priorities. In: Jamison DT, Gelband H, Horton S, et al, eds. Disease control priorities. Washington, DC: World Bank, 2017. 30212156

[19] Bilinski A Neumann P, Cohen J, Thorat T, McDaniel K, Salomon JA. When cost-effective interventions are unaffordable: integrating cost-effectiveness and budget impact in priority setting for global health programs. PLoS Med 2017; 14:e1002397. 28968399 10.1371/journal.pmed.1002397 PMC5624570

[20] Sculpher MJ, Pang FS, Manca A, et al. Generalisability in economic evaluation studies in healthcare: a review and case studies. Health Technol Assess 2004; 8:iii–iv, 1–192. 10.3310/hta8490 15544708

[21] Aliyu HB, Chuku NN, Kola-Jebutu A, Abubakar Z, Torpey K, Chabikuli ON. What is the cost of providing outpatient HIV counseling and testing and antiretroviral therapy services in selected public health facilities in Nigeria? J Acquir Immune Defic Syndr 2012; 61:221–5. 22820805 10.1097/QAI.0b013e3182683b04

[22] Scott CA, Iyer HS, McCoy K, et al. Retention in care, resource utilization, and costs for adults receiving antiretroviral therapy in Zambia: a retrospective cohort study. BMC Public Health 2014; 14:296. 24684772 10.1186/1471-2458-14-296 PMC3995515

[23] Marseille E, Giganti MJ, Mwango A, et al. Taking ART to scale: determinants of the cost and cost-effectiveness of antiretroviral therapy in 45 clinical sites in Zambia. PLoS One 2012; 7:e51993. 23284843 10.1371/journal.pone.0051993 PMC3527397

[24] Larson BA, Bii M, Henly-Thomas S, et al. ART treatment costs and retention in care in Kenya: a cohort study in three rural outpatient clinics. J Int AIDS Soc 2013; 16:18026. 23305696 10.7448/IAS.16.1.18026 PMC3536940

[25] Cianci F, Sweeney S, Konate I, et al. The cost of providing combined prevention and treatment services, including ART, to female sex workers in Burkina Faso. PLoS One 2014; 9:e100107. 24950185 10.1371/journal.pone.0100107 PMC4064981

[26] Meyer-Rath G, Miners A, Santos AC, Variava E, Venter WD. Cost and resource use of patients on antiretroviral therapy in the urban and semiurban public sectors of South Africa. J Acquir Immune Defic Syndr 2012; 61:e25–32. 22895437 10.1097/QAI.0b013e31826cc575

[27] Long L, Brennan A, Fox MP, et al. Treatment outcomes and cost-effectiveness of shifting management of stable ART patients to nurses in South Africa: an observational cohort. PLoS Med 2011; 8:e1001055. 21811402 10.1371/journal.pmed.1001055 PMC3139666

[28] Meya DB, Manabe YC, Castelnuovo B, et al. Cost-effectiveness of serum cryptococcal antigen screening to prevent deaths among HIV-infected persons with a CD4+ cell count < or =100 cells/microL who start HIV therapy in resource-limited settings. Clin Infect Dis 2010; 51:448–455. 20597693 10.1086/655143 PMC2946373

[29] World Health Organization. Global Health Observatory data repository, United Republic of Tanzania. 2016 Available at: https://apps.who.int/gho/data/?theme=main&vid=61770. Accessed 11 April 2019.

[30] Jarvis JN, Harrison TS, Lawn SD, Meintjes G, Wood R, Cleary S. Cost effectiveness of cryptococcal antigen screening as a strategy to prevent HIV-associated cryptococcal meningitis in South Africa. PLoS One 2013; 8:e69288. 23894442 10.1371/journal.pone.0069288 PMC3716603

[31] Ramachandran A, Manabe Y, Rajasingham R, Shah M. Cost-effectiveness of CRAG-LFA screening for cryptococcal meningitis among people living with HIV in Uganda. BMC Infect Dis 2017; 17:225. 28335769 10.1186/s12879-017-2325-9 PMC5364591

[32] Shiri T, Loyse A, Mwenge L, et al. Addition of flucytosine to fluconazole for the treatment of cryptococcal meningitis in Africa: a multi-country cost-effectiveness analysis [manuscript published online ahead of print 28 February 2019]. Clin Infect Dis 2019. doi:10.1093/cid/ciz163. 10.1093/cid/ciz163 PMC6912152 30816418

[33] Liechty CA, Solberg P, Were W, et al. Asymptomatic serum cryptococcal antigenemia and early mortality during antiretroviral therapy in rural Uganda. Trop Med Int Health 2007; 12:929–35. 17697087 10.1111/j.1365-3156.2007.01874.x

[34] Longley N, Jarvis JN, Meintjes G, et al. Cryptococcal antigen screening in patients initiating ART in South Africa: a prospective cohort study. Clin Infect Dis 2016; 62:581–7. 26565007 10.1093/cid/civ936 PMC4741358

